# Lung Dysfunction and Chronic Kidney Disease: A Complex Network of Multiple Interactions

**DOI:** 10.3390/jpm13020286

**Published:** 2023-02-03

**Authors:** Guido Gembillo, Sebastiano Calimeri, Valeria Tranchida, Salvatore Silipigni, Davide Vella, Domenico Ferrara, Claudia Spinella, Domenico Santoro, Luca Visconti

**Affiliations:** 1 Unit of Nephrology and Dialysis, Department of Clinical and Experimental Medicine, University of Messina, 98125 Messina, Italy; 2 Department of Biomedical and Dental Sciences and Morpho-Functional Imaging, University of Messina, 98125 Messina, Italy; 3 Unit of Nephrology and Dialysis, Ospedali Riuniti Villa Sofia Cervello, University of Palermo, 90146 Palermo, Italy; 4 Department of Biomedical Sciences and Morphologic and Functional Imaging, Policlinico “G. Martino’’, University of Messina, Via Consolare Valeria 1, 98100 Messina, Italy

**Keywords:** chronic kidney disease, lung dysfunction, pulmonary hypertension, chronic obstructive pulmonary disease, sleep-related breathing disorders, obstructive sleep apnoea, renal impairment

## Abstract

Chronic kidney disease (CKD) is a progressive disease that affects > 10% of the total population worldwide or >800 million people. CKD poses a particularly heavy burden in low- and middle-income countries, which are least able to cope with its consequences. It has become one of the leading causes of death worldwide and is one of the few non-communicable diseases where the number of related deaths has increased over the last two decades. The high number of people affected, and the significant negative impact of CKD should be a reason to increase efforts to improve prevention and treatment. The interaction of lung and kidney leads to highly complex and difficult clinical scenarios. CKD significantly affects the physiology of the lung by altering fluid homeostasis, acid-base balance and vascular tone. In the lung, haemodynamic disturbances lead to the development of alterations in ventilatory control, pulmonary congestion, capillary stress failure and pulmonary vascular disease. In the kidney, haemodynamic disturbances lead to sodium and water retention and the deterioration of renal function. In this article, we would like to draw attention to the importance of harmonising the definitions of clinical events in pneumology and renal medicine. We would also like to highlight the need for pulmonary function tests in routine clinical practise for the management of patients with CKD, in order to find new concepts for pathophysiological based disease-specific management strategies.

## 1. Introduction

Chronic kidney disease (CKD) is a global public health problem. Its prevalence is increasing worldwide and is estimated to be between 11 and 13% of the total population [1]. Kidney disease is often characterized by multiple organ dysfunction, some of which are caused by a close connection between other organs and tissues. The function of the lungs is also closely linked to the kidneys, both in health and in disease. In fact, regulation of acid–base balance, control of blood pressure and fluid homeostasis are closely linked to the interaction of the kidneys and lungs. Pathologically, the lungs can be severely compromised in CKD [2]. The prevalence of lung dysfunction, such as sleep apnoea syndrome, pulmonary hypertension and COPD (chronic obstructive pulmonary disease) is increased in these patients, regardless of the stage of the disease. The more severe the degree of kidney disease, the greater the risk of developing a pulmonary complication [3]. In addition, CKD patients often develop a restrictive spirometry pattern, related to chronic fluid overload [4]. When the glomerular filtration rate (GFR) decreases, pulmonary oedema and respiratory muscle dysfunction are more common, due to fluid retention and metabolic, endocrine and cardiovascular changes [5]. In addition, an increased incidence and prevalence of microalbuminuria (MAB) has been documented in patients with chronic lower respiratory disease. This link between kidney and lung, even in the early stages of kidney disease, suggests an important role of endothelial dysfunction in the development of lung disease. Finally, CKD also contributes to other common systemic manifestations of lung disease, such as malnutrition, muscle wasting, anaemia, osteoporosis, and cardiovascular disease [6].

**Figure 1:**
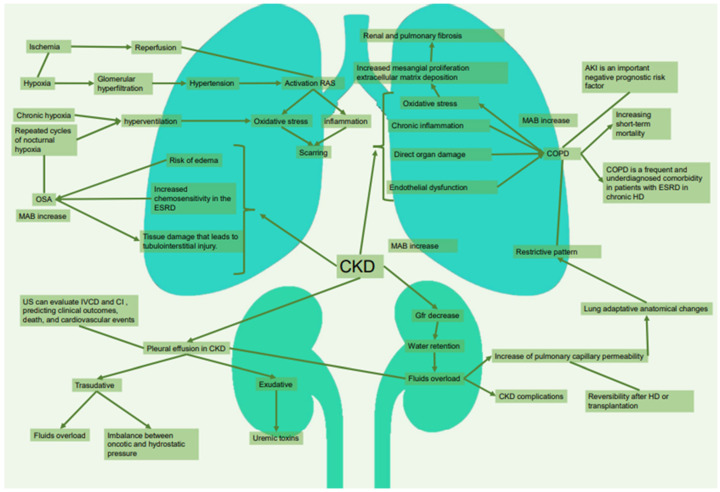
Pulmonary complications related to CKD. CKD: chronic kidney disease. RAS: renin-angiotensin System. OSA: obstructive sleep apnoea. ESRD: end-stage renal disease. IVCD: inferior vena cava diameter. CI: collapsibility index. GFR: glomerular filtration rate. HD: hemodialysis. COPD: chronic obstructive pulmonary disease. AKI: acute kidney injury. MAB: microalbuminuria.

The aim of this review is to describe the co-existence of renal and pulmonary disease and to highlight the need for pulmonary function testing in routine clinical practise for the management of patients with CKD (Figure 1).

## 2. Chronic Obstructive Pulmonary Disease (COPD) and CKD

COPD is a chronic and progressive disease characterized by persistent respiratory symptoms and airflow limitation (AFL) due to the inflammation of the airways and/or chronic alveoli, causing parenchymal changes that are not fully reversible. The most frequent respiratory symptoms include dyspnoea, cough, sputum and wheezing. Spirometry is fundamental for the diagnosis of COPD. The presence of an FEV1/FVC (forced expiratory volume/forced vital capacity) ratio < 70% is a diagnostic of airflow limitation. The severity of these airflow limitations determines the staging (Table 1).

The estimated prevalence is approximately 384 million people worldwide, and COPD is expected to be the third leading cause of death worldwide by 2030 [7]. COPD is increasingly occurring in patients with CKD. On the contrary, CKD is already a well-known comorbidity in COPD patients. Unfortunately, this association may go unrecognized. Most of these patients are elderly and frail. They may have normal serum creatinine concentrations due to malnutrition, and a reduction in muscle mass, making the eGFR formula for kidney damage ineffective. There are numerous pathophysiological mechanisms that contribute to this association, and many risk factors are common to the two diseases, such as older age, smoking and higher levels of inflammatory markers [8]. Cigarette smoking is a major cause of COPD and impairs kidney function. Nicotine is responsible for increased oxidative stress, which leads to increased mesangial proliferation and deposition of the extracellular matrix, contributing to the development of renal and pulmonary fibrosis. In addition, COPD and CKD also share important features of premature ageing and systemic inflammation. Novel inflammatory biomarkers [9,10] suggest that CKD causes high levels of chronic inflammation, which can affect the lungs and airways causing direct organ damage or inducing the development of endothelial dysfunction contributing to the strong association between COPD and CKD [11].

Numerous studies show an association between these two diseases, and it is not always possible to distinguish which is the primary disease (Table 2).

CKD represents not only a comorbidity of COPD but is also an independent factor for the exacerbation of COPD and increased mortality in COPD patients. In fact, CKD patients have lower pulmonary function, respiratory and peripheral muscle strength values compared to the general population, which reflects negatively in the quality of life [27].

Finally, the prevalence of COPD seems to be increased in patients with ESRD on chronic HD, but the literature on the topic is still scarce. Therefore, a lung function test should be performed in all HD patients at increased risk.

## 3. Pulmonary Hypertension in CKD

It is increasingly recognised that pulmonary hypertension (PH) may also be another very common and important condition in patients with CKD. Experimental studies suggest that activation of the RAAS occurs earlier in the course of PH. Vascular congestion in the kidneys, due to increased central venous pressure, leads to a reduction in renal perfusion pressure and glomerular filtration rate [28]. The underlying aetiology and optimisation of volume status are central to PH management in patients with CKD. PH is defined as a mean pulmonary arterial pressure, >20 mmHg at rest [29]. PH classification includes five groups based on patient presentation, pathophysiology, and therapeutic strategies [30]. Pulmonary arterial hypertension (PH1) is characterized by vasoconstriction and remodelling of the pulmonary vascular district, leading to vascular hyperplasia and increased flow resistance [31]. This process also sometimes leads to renal involvement due to decreased cardiac output, renal venous congestion and decreased renal function with the risk of increased mortality. The prevalence of CKD in this group ranges from 4 to 36% [32]. Treatment is usually aimed at limiting the action of vasoactive molecules involved in the development and progression of PH1, such as prostacyclin and nitric oxide. PH2 is secondary to left heart disease and is the main form associated with CKD [33]. The increased venous pressure leads to a disruption of the alveolar-capillary interface, increased endothelial permeability and damage to the capillary walls [34]. Pulmonary hypertension can exacerbate many obstructive pulmonary diseases (PH3). Several studies have highlighted the increased incidence of CKD in COPD patients and the increased mortality when PH3 and CKD are present simultaneously [35]. Group 4 of PH is not yet well correlated with CKD. It includes forms of PH due to chronic thromboembolism, and is characterized by small vessel changes, resulting from the organization and consolidation of thrombi created by acute thromboembolic events [36]. PH5 includes a heterogeneous spectrum of pathologies that cannot be classified in the other groups. This includes renal failure, which is classified as a systemic disease, due to its pathogenic properties [30]. This classification shows that patients with renal disease are more frequently affected by PH than the general population. Several studies have investigated the prevalence and outcomes of PH in CKD patients on conservative therapy, and in patients requiring dialysis. Among the characteristics of CKD, anaemia and older age had a greater risk of association with PH, as did hypertrophy, or a reduced left ventricular excretory fraction. Therefore, there was a directly proportional relationship between the two pathologies, with the incidence of PH increasing with worsening CKD, occurring more frequently in dialysis patients [37,38]. One meta-analysis reported a 30% prevalence of PH in patients with CKD, which was even higher in the ESKD subgroup (35%) [39]. A recent study with a large sample (30,052 CKD patients with PH) confirmed that PH was associated with a higher risk of death during the five-year follow-up period (HR 1.47, 95% CI, 1.40–1.53). In addition to mortality, PH patients had an increased risk of hospitalization, mainly due to cardiovascular causes (rate ratio 4.61) [40].

As mentioned earlier, CKD alone can contribute to the development of PH due to changes in the vasculature (stiffening and vascular calcification) caused by the pro-inflammatory state [41]. Indeed, the release of uraemic toxins leads to an alteration in vasoregulation, with direct dysfunction of the pulmonary circulation [42]. Alterations in the metabolism between calcium and phosphorus may also increase the risk of developing vascular calcification and increased pulmonary resistance [43]. This was also highlighted in one of the most important clinical trials, assessing risk factors for progression of CKD, the Chronic Renal Insufficiency Cohort (CRIC) study. About 21% of 2959 patients with CKD (without dialysis) had PH, the presence of which correlated with an increased risk of cardiovascular events and mortality [44]. Patients with end-stage renal disease who undergo HD are often affected by respiratory insufficiency associated with interstitial disease, vascular congestion, leukocyte infiltration and haemorrhagic alveolitis [45]. PH is consistently associated with adverse outcomes, including all-cause mortality and cardiovascular events, in patients with advanced kidney disease. Risk stratification of CKD and ESRD could consider PH as a significant predictor for long-term survival [46]. In addition, an arteriovenous fistula (AVF) can cause haemodynamic effects, decrease systemic vascular resistances, and increase venous return and cardiac output, thus contributing to the development of PH [47]. AVF blood flow, which is higher in HD patients than in PD patients who previously had AVF, has been shown to significantly affect systolic pulmonary arterial pressure (SPAP) [48]. Other factors consistent with the development of PH in HD patients are related to the composition of dialysis membranes or the possibility of microembolism formation due to small bubbles in the dialysis circuit [49]. All patients with PH-related signs or symptoms should undergo diagnostic screening. Carl P. Walter et al. suggested the possibility of using a diagnostic algorithm for the identification and management of PH in patients with CKD and on dialysis [50]. Echocardiographic examination plays an important role and must be performed as soon as it is optimised for the patient’s volume. In HD patients, this examination should be performed in the post-dialysis period to obtain better data. At this time, assessment of tricuspid regurgitation velocity (TRV) and association with PH signs will classify the likelihood of PH as low, intermediate, or high [30]. From this point on, interdisciplinary assessment with the cardiologist and pulmonologist is essential to determine whether left heart disease or chronic respiratory disease with hypoxia may be contributing to the development of PH, and to optimise the therapeutic strategy. A recent meta-analysis analysed the influence of PH in kidney transplantation (KT) patients. The authors demonstrated that patients were at higher risk of death, delayed graft function, or graft failure. In fact, PH might represent an extensively available and valuable tool for risk stratification in KT patients. These findings support the routine assessment of PH in patients on the KT waitlist [51]. Studies examining pre-existing PH as a predictor of adverse events after KT are limited to observational data, and more specific studies should be conducted in the future.

## 4. Sleep-Related Breathing Disorders in CKD

It is estimated that about 1 billion people in the general population are estimated to have sleep-related breathing disorders (SBD) [52]. The most common SBD is sleep apnoea (SA). It is recognized worldwide as an independent risk factor for cardiovascular and cerebrovascular morbidity and mortality [53]. SA can be defined as a consecutive reduction (hypopnea) or interruption (apnoea) of breathing during sleep with resulting hypoxemia. The number of episodes of apnoea and hypopnoea during sleep is quantified by the apnoea-hypopnea index (AHI), which classifies sleep apnoea into three categories (Table 3).

In addition, SA is divided into two types depending on the origin of the disorder:

- Obstructive sleep apnoea (OSA) occurs due to intermittent closure of the upper airway during sleep.
- Central sleep apnoea (CSA) occurs due to intermittent loss of respiratory drive.

A proportion of patients appear to have features of both types (OSA and CSA), a condition often referred to as mixed apnoea [55].

CKD patients have a significantly higher prevalence of sleep apnoea than the general population. It is estimated that 50–80% of these patients suffer from sleep-disordered breathing, compared to 2–4% of the general population or 20–30% of patients with other co-morbidities such as diabetes or heart failure [56,57,58]. The risk of developing SA increases progressively with decreasing GFR, contributing to the loss of renal function and increased cardiovascular risk. Volume expansion associated with excess fluid is a common complication in the moderate and severe stages of CKD. This is one of the main risk factors for the occurrence of OSA in this population, as the presence of oedema can block the upper airway due to a fluid shift in the rostral area [59]. In patients with ESRD, increased fluid overload predicts the severity of sleep apnoea, and intensification of renal replacement therapy or increased fluid removal by ultrafiltration attenuates the severity of sleep apnoea. The changes in metabolic balance that occur in CKD lead to an increased chemoreflexive responsiveness to hypercapnia, which lowers PaCO2 below the eupnoea threshold and contributes to the pathophysiology of sleep apnoea [60]. In a study of 58 individuals with ESRD, it was shown that those with sleep apnoea (AHI > 10) had augmented responsiveness of both the peripheral and central chemoreflexes compared with those without OSA, suggesting that subsequent ventilatory instability caused sleep apnoea in this population [61]. It is not known what specific ESRD-related factors contribute to the increased chemoreflex responsiveness in people with CKD. Some authors have suggested that metabolic acidosis or uraemia may cause increased responsiveness. However, Beecroft et al. reported increased chemosensitivity in the ESRD study population without metabolic acidosis, suggesting that other factors play a role [62]. For example, there are other mechanisms of damage that may exacerbate CKD in patients with SA, due to tissue damage leading to tubulointerstitial injury. These are represented by both glomerular hyperfiltration and the state of chronic hypoxia [63], caused by SA directly by hypoxia and indirectly leading to hypertension and activation of the renin-angiotensin system with resulting inflammation and oxidative stress [64,65]. Specifically, chronic hypoxia is the main mediator of progressive scarring of the renal parenchymal with subsequent impairment of renal function. Repeated cycles of nocturnal hypoxia followed by hyperventilation create a situation similar to that of ischaemia-reperfusion injury, resulting in tissue damage due to the excessive production of reactive oxygen species and inflammation during reoxygenation [66,67]. Altered oxidative balance and systemic inflammation are mediators in the pathogenesis of endothelial dysfunction and deterioration of renal function [68]. A recent study in a rat model showed that intermittent hypoxia causes hyperplasia of glomerular mesangial cells, oedema of tubular epithelial cells and loss of the brush border of renal cells [69]. Together, these processes can cause structural and functional damage to the kidney, leading to CKD [70,71,72,73]. Hypoxia also causes renal tubule cells to undergo epithelial-to-mesenchymal transformation and fibroblast activation, leading to interstitial fibrosis and damage to peritubular capillaries [74]. Chronic hypoxia also causes defects in the mitochondrial cells of the renal tubules with subsequent activation of apoptosis. All these processes lead to degeneration of the renal tubules. In a study of 31 patients with OSA and 13 control subjects, Zalucky et al. [75] found that patients with OSA and severe hypoxia had higher RAS activity than patients with moderate hypoxia and control subjects in a dose-dependent manner. Furthermore, the severity of hypoxia was not associated with the response of the BP or the systemic circulating RAS component to angiotensin II, suggesting a direct effect on renal RAS activation [75]. Hypertension is one of the most common causes of CKD, and RAS inhibition represents a key therapy for patients with renal impairment. Hypertension can damage the kidneys through various pathological mechanisms, including diffuse glomerulosclerosis, mesangial hypertrophy, nephrosclerotic glomerulonephropathy, glomerular fibrosis, and interstitial renal fibrosis [76,77]. The role of OSA in causing hypertension is now well established by experimental studies in animals and epidemiological studies in humans [78]. The main mechanism is the effect of repeated cycles of hypoxia/hypercapnia leading to sympathetically mediated vasoconstriction [79]. Wake-up calls that terminate apnoeas also lead to an increase in sympathetic activity [80]. OSA can also increase the stiffness of the arterial walls, which can damage the kidneys, causing microvascular damage and ischaemia of the renal tissue [81,82]. As renal disease progresses to its end stage, the onset of uraemic neuropathy contributes to the worsening of SA by increasing upper airway collapse [83]. Despite the higher prevalence of SBD (sleep breathing disorders) in the overall stages of CKD to ESRD, it remains underestimated as patients with CKD or ESRD have fewer symptoms of SDB such as snoring, witnessed apnoea, daytime sleepiness, non-restorative sleep and morning headaches compared to those without CKD [84]. Interestingly, the clinical picture of patients with concurrent OSA and ESRD differs in several ways. ESRD patients with OSA have a lower BMI and neck circumference than OSA patients without kidney disease. Furthermore, ESRD patients often suffer from daytime sleepiness, due to poor sleep quality from the kidney disease itself or from other sleep disorders such as insomnia and periodic leg movements that may mask the presence of OSA [85]. In the later stages of CKD, when patients require renal replacement therapy/dialysis, there is an increased prevalence, and severity of sleep apnoea is evident [86,87]. Nicholl et al. reported that prevalence of sleep apnoea increases as CKD progresses and GFR decreases, rising to 60% in ESRD, and this increased prevalence is not explained by age, gender, BMI, or the presence of cardiovascular disease [88]. In two trials, Tang and Hanly showed that intensification of dialysis treatment attenuates the severity of sleep apnoea in ESRD patients [89,90]. In addition, a meta-analysis of nine studies examining the effects of renal replacement therapy on sleep quality and disturbance found that intensive renal replacement therapy reduced the Apnoea Hypopnea Index (AHI) compared with conventional renal replacement therapy (OR, 0.66; 95% CI, 0.51–0.84; *p* < 0.001) [91]. In an interventional study of 15 patients with sleep apnoea and ESRD undergoing conventional HD, which included subjects with both OSA and CSA, the additional removal of 2.2 l of fluid during a single ultrafiltration session resulted in a 36% reduction in AHI, with no changes in uraemic or metabolic status. The degree of reduction in AHI correlated with the decrease in total body extracellular fluid volume (r^2^ ¼ 0.322; *p* ¼ 0.027) [92]. The bidirectional relationship between obstructive sleep apnoea and renal disease is also evident in the response to therapy. In a study by Nicholl et al., researchers found that treatment of OSA with CPAP resulted in an overall improved kidney health, as measured by the decreased renin-angiotensin system activity and reductions in mean arterial pressure, plasma aldosterone, and urinary protein excretion [93]. Conversely, treating ESRD patients with nocturnal HD had a similar beneficial effect on patients with sleep apnoea. A study of fourteen patients who underwent nocturnal HD sessions showed a significant reduction of apnoeic events and an increase in minimum oxygen saturation. Other studies reported similar effects of CPAP on kidney haemodynamics by decreasing hyperfiltration, reducing filtration fraction, increasing renal blood flow, and slowing renal damage [94]. These findings demonstrate a key role of fluid overload in the pathogenesis of sleep apnoea in ESRD and show that fluid deprivation attenuates sleep apnoea without altering uraemic status. The exact mechanisms by which fluid withdrawal works remain to be elucidated but include improved UA mechanics and/or improved stability of ventilatory control. Finally, the extent to which uraemia independently contributes to the pathogenesis of SA has not been thoroughly investigated. All these results indicate that fluid overload plays an important role in the development of sleep apnoea in ESRD. However, further studies are needed to better understand the effects of fluid overload on important pathophysiological mechanisms, such as airway collapsibility and airway instability. This research may ultimately enable a personalised approach to the treatment of ESRD sleep apnoea as an alternative to CPAP by optimising fluid status and tailoring and nuancing modifications to renal replacement therapies.

## 5. Microalbuminuria (MAB) in Chronic Lower Respiratory Diseases (CLRDS)

MAB is expressed by the presence of albumin in the urine, which is normally undetectable by conventional semi-quantitative tests. It is considered an early marker of kidney damage and is associated with an increased progression of kidney disease. It is also an independent risk factor for the development of cardiovascular disease [95]. The National Kidney Foundation defines MAB as the urine albumin creatinine ratio (UACR) between 20 mg/g for men and 30 mg/g for women and the upper limit of 299 mg/g for both sexes [96]. The normal glomerular capillary membrane, consisting of 5 nm wide pores with a negative surface electrical charge, prevents the passage of albumin, a negatively charged macromolecule (molecular weight 69.000 and radius 3.6 nm), except for 0.1%, which is almost completely reabsorbed at the level of the proximal tubules. MAB is an expression of increased permeability in the glomeruli, which is generally secondary to microvascular damage and is therefore considered an early marker of endothelial dysfunction. Several factors are involved in the pathogenesis of this disease. In pathological conditions such as diabetes and arterial hypertension, it is a known event with a variable prevalence (20–30% in diabetics, 5–40% in hypertensives) [97]. Patients with CLRDS, which includes chronic obstructive pulmonary disease (COPD), chronic bronchitis, emphysema and asthma, have systemic inflammation, hypoxaemia, increased sympathetic activation and increased aortic stiffness, which may contribute to endothelial dysfunction. For this reason, the co-existence of MAB is not a rare event [98]. Oelsner et al. analysed six population-based cohort studies to investigate the association between albuminuria and pulmonary dysfunction. The 11,911 participants had a mean age of 60 years, of whom 51% had never smoked and 11% were current smokers. UACR was measured in random samples and lung function was assessed by spirometry. The results of the study showed that MAB was associated with lower lung function, accelerated deterioration of lung function and an increase in COPD and COPD exacerbations. Furthermore, these associations were independent of smoking, diabetes and hypertension [99]. Yoon et al. used data from the 5th Korean National Health and Nutrition Examination Survey, which included 6020 participants (2643 men and 3377 women), assessed lung function, and measured urinary albumin. The results showed that restrictive and obstructive patterns were higher in the MAB group than in the no MAB group (restrictive patterns: 20.1% vs. 10.2%, *p* < 0.001 in men; 14.2% vs. 9.3%, *p* = 0.004 in women; obstructive patterns: 27.3% vs. 22.5%, *p* = 0.002 in men; 8.8% vs. 6.0%, *p* = 0.033 in women). The percentage predicted value of FVC was higher in the group without MAB than in the group MAB in both sexes (mean 6 SD: 91.8% 611.3 vs. 87% 612.2, *p* < 0.001 in men; 93.6% 611.5 vs. 91.1% 612.1, *p* < 0.001 in women). The percentage predicted value of FEV1 in the group without MAB was also higher in men than in the MAB group (mean 6 SD: 89.8% 613.4 vs. 86.6% 614.5, *p* < 0.001 in men), but not in women [100]. Several studies have demonstrated the co-existence of MAB and COPD. Casanova et al. studied 129 patients with stable COPD, compared with 51 smoking controls. They showed that COPD patients had significantly higher levels of MAB than smokers without obstruction (median 8 vs. 4.2 mg/g; *p* < 0.001). The absolute levels of MAB in COPD patients were 8.1 mg/g (2.9–114) 4.1 mg/g (1.7–24.4) in control subjects (*p* < 0.001) and 23 vs. 4.5% (*p* < 0.008) as a percentage of patients reaching the pathological threshold. The difference between the two groups remained significant, even when patients with diabetes and hypertension were excluded (*p* < 0.001) [101]. Tibet et al. studied twenty-five hospitalised cases with acute exacerbation of COPD compared with 25 healthy subjects. MAB was detected in 14 (56%) subjects on admission and in seven (28%) subjects on discharge in the COPD group and in one (4%) subject in the control group, with statistically significant differences between these groups (admission control *p* < 0.001, discharge control *p* = 0.023, admission-discharge *p* = 0.016). MAB was related to hypoxaemia but not age, arterial pH, pCO2, FEV1 percent predicted, FVC percent predicted and FEV1/FVC, and has no predictive role for mortality [102]. In a 12-year follow-up study, Romundstad et al. investigated the association between MAB and COPD in 3129 participants. They showed that the risk of microalbuminuria increased significantly at lower FEV1% levels (*p* = 0.001). In addition, COPD patients with microalbuminuria had higher all-cause mortality (hazard ratio 1.54, 95% CI 1.16–2.04) compared with COPD patients without microalbuminuria [103]. Kaysoydu et al. [104] studied seventy COPD patients compared to 40 healthy volunteers. They found that the mean MAB value was statistically significantly higher in COPD patients (*p* < 0.001). In addition, the presence of MAB in these patients was associated with higher levels of C-reactive protein (CRP) and a higher value of nocturnal pulse pressure, compared to COPD patients without MAB.

Recently, Mendy et al. [105] showed that albuminuria is associated with subsequent mortality from CLRD, as well as from influenza and pneumonia, independent of diabetes or CKD. They analysed data from the National Health and Nutrition Examination Survey (NHANES III) and found that a 10-fold increase in albuminuria was associated with an 88% higher risk of death from CLRD (HR, 1.88; 95% CI, 1.25–2.84). Similarly, a 10-fold increase in albuminuria was associated with a 103% increase in the risk of death from influenza and pneumonia (HR, 2.03; 95% CI, 1.31–3.16). MAB may also be present in patients with intermittent hypoxaemia, due to obstructive sleep apnoea syndrome (OSAS). Bulcun et al. evaluated 98 consecutive patients with OSAS and 26 non-apnoeic snorers. MAB was found in 25 patients with OSAS, and only in one non-apnoeic snorer. Thus, the presence of microalbuminuria was significantly higher in OSAS than in non-apnoeic snoring subjects (*p* < 0.05) and it was associated with a higher AHI (*p* = 0.0001), desaturation index (DI) (*p* = 0.0001) but a lower minimum O_2 value_ (*p* = 0.0001). In the linear regression model, there was a positive relationship between MAB and desaturation index (*p* = 0.0001), while there was a significant negative relationship between MAB with minimum O (*p* = 0.003) [106]. Tsioufis et al. compared 62 untreated hypertensive OSA patients with 70 hypertensive non-OSA patients. They found that MAB was greater in OSA subjects (57%) than in non-OSA subjects (*p* < 0.001). In the multivariable linear regression analysis, they showed that the independent predictors of MAB were AHI (*p* < 0.001) and 24-hour pulse pressure (*p* = 0.01) [107]. In an observational study, 496 OSAS patients underwent polysomnography and urine collection. Adjusted linear mixed-model analyses showed that higher AHI was significantly associated with higher levels of MAB (*p* < 0.006), indicating that OSAS is significantly associated with increased urinary albumin excretion, especially in patients with more severe disease [108]. A recent meta-analysis, including six studies examined the effectiveness of CPAP treatment in reducing MAB in OSA patients. The results indicated that CPAP therapy had a positive effect on reducing MAB in these patients, suggesting that early intervention with CPAP could help prevent the occurrence of CKD and cardiovascular events and then improve outcomes [109]. In contrast, there is less evidence of MAB in pulmonary hypertension, although this association cannot be excluded. In fact, Nickel et al. evaluated a total of 283 patients (two independent cohorts) diagnosed with pulmonary arterial hypertension, compared to 68 healthy controls. The results showed that the group with pulmonary arterial hypertension had significantly higher levels of MAB, compared to the healthy controls (*p* < 0.01). In addition, higher levels of MAB were significantly associated with older age, lower six-minute walk distance, increased CRP levels and poorer treatment outcomes [110].

## 6. Fluid Overload and Lung Congestion in CKD

Fluid overload is a common problem leading to severe complications in CKD patients. This is due to the tendency for water retention due to a decreased glomerular filtration rate. Fluid overload and a possible increase in pulmonary capillary permeability promote functional and anatomical pulmonary adaptation changes leading to a restrictive spirometric pattern (defined as FEV1/FVC ≥ 0.70 and FVC% < 80). In a recent study of patients referred for right heart catheterisation at an academic medical centre, Edmonston and colleagues reported that isolated postcapillary and combined pre- and postcapillary PH were more common than isolated pre-capillary PH or pulmonary arterial hypertension in patients with CKD and renal failure [41]. Mukai et al. [111] analysed lung function by spirometry in 404 individuals at different stages of CKD. The results showed an increased prevalence of restrictive lung disease in patients with CKD, especially in stage 5 patients with a GFR < 15 mL/min (36%). In ESRD patients receiving maintenance therapy HD, restrictive lung disease is the most common pulmonary dysfunction. In addition, patients with restrictive airway disease have been shown to be at increased risk for ESRD [112].

Pleural effusion is another typical pulmonary complication of CKD. It can be divided into transudative pleural effusion (low content of proteins and/or cells), which is mainly caused by fluid retention due to cardiovascular disease, or an imbalance between oncotic and hydrostatic pressure, as generally occurs in nephrotic syndrome, and exudative pleural effusion (high concentration of proteins and/or cells), which is caused by direct exposure to uraemic toxins.

In a recent study, Jabbar et al. [113] examined 280 CKD patients, most of them stage 4 and 5, and showed that pleural effusion is a common feature in these patients. In fact, 212 (75.7%) patients had transudative pleural effusion due to fluid overload, with heart failure (HF) being the most common cause, while 68 (24.3%) patients had exudative pleural effusion, with tuberculosis being the most common cause.

In a prospective study, Ray et al. [114] investigated the presence of pleural effusions in 430 CKD patients (stage 3–5). They found a prevalence of 6.7% (29 patients) in this cohort. Exudates and transudates were found with equal frequency. HF was the most common cause of transudative pleural effusions (41.9%), while tuberculosis (25.8%) and uraemic effusions (19.4%) were the main causes of exudative pleural effusions. In addition, in CKD patients who underwent autopsy, pleural effusion was found in 20–40% of cases [115]. Patients receiving maintenance therapy HD have an increased risk of developing pleural effusion. Shaik et al. [116] conducted a prospective cohort study of 250 patients on maintenance therapy HD. They investigated the presence of respiratory disease in these patients. The result showed that seventy-nine patients (31.6%) had significant respiratory symptoms such as dyspnoea, cough and chest pain. Pleural effusion was present in 23 patients (9.2%) and was the most important pulmonary complication in this population. Fluid overload thus remains a current problem in renal patients, especially in ESRD patients undergoing HD treatment. Fluid overload assessment represents a particularly important challenge for those who daily practice with CKD patients.

Radiology still represents a non-renounceable mean for the evaluation of fluid sequestration, but some heavy prices must be considered during radiologic assessment. First of all, it is a not an immediate evaluation and may take hours before yielding data useful for clinical workup; in addition, it requires moving the patient to radiology rooms (x-ray or CT), which, in delicate clinical conditions, may represent a risk. Finally, cumulative radiation dose over time must be considered, as such evaluations need to be repeated with a certain frequency.

Today, a precious role is played by ultrasound which is demonstrated to be reliable, with a quick execution and with the advantage of a bedside evaluation.

## 7. Imaging of Pulmonary Dysfunction in CKD

The haemodynamic and biohumoral link between CKD and lung function is a bidirectional pathway that constantly influences clinical management. This link should therefore not be underestimated in radiological assessment. Pulmonary signs alone are indicative of pathology and usually require specific measures, whereas in the radiological assessment of CKD, some signs must be considered paraphysiological. On the other hand, depending on the stage of CKD, intermediate complications need to be identified immediately.

Radiological examination provides a reliable overview of the condition of the lungs, which may appear, due to CKD.

### 7.1. Pulmonary Fluid Overload—Conventional Radiology

An increase in hydrostatic pressure indicates progression of the disease, which is positively related to the signs visible on the CXR.

Early signs of interstitial congestion at CXR are seen in progressive vasodilation and cranial redistribution of blood flow [117]. A widening of the upper mediastinal shadow and an increase in cardiac shadow volume may also be noted, especially in HF.

A further increase in hydrostatic pressure determines interstitial involvement, which is evident on radiography by a progressive peribronchovascular cuff, due to central interstitial fluid filling and the presence of peripheral interstitial lines associated with the B-Kerley lines (millimetric peripheral horizontal lines, indicating thickening of the interlobular interstitial septa) or the A-Kerley lines (oblique lines running from the peripheral fields to the hila and representing lymphatic congestion).

Progression of interstitial involvement leads to the filling of the alveolar space with airspace opacities [118].

Manifest pulmonary oedema typically affects both lungs and a variety of patterns are known, from bat-wing shaped pulmonary oedema to a diffuse pattern with patchy opacities. The confluence of opacities results in extensive opacities that tend to consolidate.

In rare cases, pulmonary oedema can also occur unilaterally. This particular phenomenon, associated with cardiac causes (e.g., mitral valve dysfunction or pleural expansion), can mislead physicians and requires a precise differential diagnosis to distinguish it from other causes, such as infections and cancer [119].

Vascular congestion is better visible with chest CT and interstitial thickening of the interlobular septa in both the lower and upper lobes and is an early sign of interstitial fluid overload. The filling of the alveolar spaces can be seen on imaging at CT with the typical ground-glass appearance.

The combination of alveolar fluid filling and inflammatory damage to the alveolar wall leads to the consolidation of the air space. Inflammatory-immune alveolar damage usually presents as diffuse damage with rapid progression in acute respiratory distress syndrome (ARDS); in diffuse alveolar damage, all lung fields are affected by diffuse, patchy consolidations [120].

### 7.2. Pulmonary Fluid Overload—Ultrasound (US)

In recent years, ultrasonography has begun to play an important role in determining fluid homeostasis.

Chest US allows direct pulmonary assessment of fluid overload.

The disappearance of physiological A-lines, due to the presence of ring-down artefacts, also known as B-lines, represents its main sign. The B-lines can be compared to the B-Kerley lines seen on an X-ray examination of the lungs. Indeed, both represent thickening of the interlobular septa on different imaging modalities [121].

It should be noted that ultrasonography allows the assessment of pulmonary fluid overload at the preclinical stage and is now considered an emerging tool for monitoring fluid accumulation in HD patients [122,123]. However, those who routinely use chest ultrasound in HD patients must remember that B-lines are not a pathognomonic sign of watery interstitial filling, but a general sign of thickening of the interstitial septa, and that an accurate differential diagnosis with interstitial lung disease must be considered (e.g., note that fibrotic B-lines are diuresis resistant).

Mallamaci et al. [123], in a study of 75 HD patients (26% of whom had NYHA class HF III-IV), showed that US chest radiography had good agreement between observers and probes. In this study, up to 57% of asymptomatic HD patients had moderate-severe congestion.

In their meta-analysis, Maw et al. [124] investigated the prognostic value of chest US for clinical outcomes in patients with cardiogenic pulmonary oedema. They demonstrated that point-of-care lung ultrasonography is more sensitive than chest radiography (CXR) in detecting pulmonary oedema in acute decompensated HF.

However, the impact of chest US in HD patients is under discussion.

Zoccali et al. [125] highlighted the importance of US lung ultrasound for prognostic data related to death and cardiovascular risk factors highlighting that 71% of patients with moderate/severe pulmonary congestion were asymptomatic or had mild symptoms HF. However, in a recent randomised-controlled trial by the group [126], enthusiastic results on death risk reduction from previous studies have been revised.

In this randomised-controlled trial on HD patients with high cardiovascular risk and large concomitant cardiovascular disease, the authors guided their ultrafiltration and HD strategy using pulmonary US and successfully and safely reduced pulmonary congestion in the active arm of the study. This strategy was no more effective in reducing the composite endpoint (time to death or myocardial infarction or decompensation) than the usual care strategy HF.

In the study LUS-HF [127], the authors examined the composite primary endpoint of urgent visit hospitalisation for the exacerbation of HF, and death in 123 patients admitted for HF. Patients were randomised to receive either standard post-treatment or US-guided diuretic therapy.

Patients in the active arm had a 48% risk reduction for a composite endpoint that included mortality, time to urgent visit and hospitalisation for the exacerbation of HF, but mortality did not differ between the two groups.

In another study of 244 patients with chronic HF [128], randomised to US-guided pulmonary therapy or physical examination alone, in addition to physical examination, there was a significant reduction (56%) in the risk of hospitalisation for the acute decompensation of HF, but again, no difference in mortality was observed between the two study arms.

The results of these two studies inspired Zoccali et al. to conduct a post-hoc analysis for the LUST study [129]. They observed a risk reduction for recurrent episodes of decompensated HF and cardiovascular events in the lung-controlled group. The differences observed in this analysis remain controversial and further RCTs and meta-analyses are needed to confirm the results.

Overall, these data suggest that the use of US thoracic examination can improve prognosis in HD patients. Thus, important evidence is accumulating that US chest examination is a useful adjunct for the diagnosis and management of fluid overload in CKD patients.

US may also be used to assess the circulating fluid volume. A smart technique to evaluate intravascular circulating volume is the diameter of the inferior vena cava (IVCD) and its collapsibility index (CI). Normal values of the CI range from 0.75 to 0.40 and correlate with right atrial blood pressure, which is considered the reference value. IC values above 0.75 reflect overhydration, while IC values below 0.40 indicate dehydration [130]. In their study on 89 chronic HD patients, Brennan et al. [131] measured the IVCD diameter immediately before and within 30 minutes after each dialysis session and, when possible, at the onset of intradialytic symptoms such as cramps, chest pain or hypotension. The results showed that hypovolaemic patients detected by inferior vena cava ultrasound had more episodes of chest pain and cramps and more episodes of hypotension. On the contrary, there was a poor relationship between dry weight targets and IVC collapsibility.

Katzarski et al. [132] studied IVCD to assess fluid status and dry weight after HD in 35 patients. The IVCD diameter was assessed before and 35 to 40 minutes after HD. The results showed that the IVCD value at the end of HD was below the reference range. However, in the following 1 to 2 hours, the IVCD value increased to values above the intravascular space and, in some cases, even to values above the reference range. IVCD measured at the end of or shortly after HD can therefore be misleading in the assessment of dry weight. In conclusion, US assessment of VCI is a useful tool for assessing volume status in the general population but is less reliable for determining dry weight in patients with CKD, and especially in patients undergoing HD treatment [133].

### 7.3. Imaging of COPD in CKD

CKD patients may develop airway changes due to inflammatory biomolecules. Therefore, COPD must be considered as a possible comorbidity in any patient with CKD, especially if cigarette smoking is considered a higher risk factor.

COPD-related lung changes initially affect the bronchial tree and become visible on X-ray only when the pathology becomes clinically apparent. CXR signs of COPD are increased transparency of the parenchymal fields due to hyperinflation, flattening of the diaphragm, and attenuation or absence of normal vascular branches [134]. Chest CT offers a deeper assessment of empysema changes during COPD; in fact, irreversible changes associated with the destruction of the septal wall can be divided into three main types according to the pathological pattern of destruction of the alveolar wall.

- Centrilobular emphysema, strongly associated with cigarette smoking, is associated with the loss of pulmonary bronchioles, relative sparing of distal alveolar walls and is usually localised in the upper lobes of the lungs (particularly the posterior regions).
- Panlobular emphysema is associated with the extensive loss of alveolar septa, is found predominantly in the lower lobes of the lungs and is associated with Alpha-1-antitrypsin.
- Paraseptal emphysema typically affects the subpleural alveolar spaces and shows a characteristic peripheral pattern in the upper lung. It is associated with spontaneous pneumothorax.
- Paracicatricial emphysema involves the air spaces around the scarred lung and is characterised by the distorted scar expansion of the air spaces [135].

Matsuoka et al. [136] showed an inverse relationship between emphysema changes and the cross-sectional area (CSA) of distal vessels (vessel with an area of <5 mm^2^); this finding demonstrates how permanent changes in COPD with airway destruction involve vascular changes, leading to a reduction in the total vascular pulmonary cross-sectional area. Reduced compliance of small pulmonary vessels due to remodelling of subsegmental pulmonary vessels also leads to statistically significant changes in vascular resistance. In another study, Matsuoka et al. [137] showed a significant negative correlation between the reduction in distal vascular cross-sectional area and the pulmonary arterial pressure measurement on right heart catheterisation in patients with severe emphysema (r = −0.512, *p* < 0.0001). According to the authors, the correlation was influenced by the degree of muscular arterial endothelial dysfunction rather than the degree of emphysema.

### 7.4. Imaging of Pulmonary Hypertension in CKD

CKD is often associated with pulmonary hypertension, which has significant implications for prognosis and therapeutic management, and must therefore be considered for differential diagnosis during routine radiological examination.

CXR may show signs of PH, such as dilatation of the pulmonary boot and enlargement of the vascular hila; however, the absence of these signs does not exclude PH. In the case of heart failure PH (group II according to the clinical classification ESC), enlargement of the ventricles may also be seen on CXR.

Echocardiography is a globally available technique that plays an important role in the diagnosis of PH. Ventricular morphology and function, valvular dysfunction and haemodynamic parameters can be accurately measured; it also provides valuable information on the aetiology and response to therapy in group 2 PH [138,139].

Echocardiographic assessment requires multiparametric evaluation to confirm the suspicion of PH: an estimate of systolic pulmonary arterial pressure can be obtained by peak tricuspid regurgitation velocity (TRV). Indeed, a peak TRV of 2.8 m/s must raise suspicion of PH. However, at least two of the additional echocardiographic signs suggestive of pulmonary hypertension are required to make the diagnosis.

Echocardiographic signs of right ventricular overload/malfunction may include: RV/LV basal diameter/area ratio > 1.0, flattening of the interventricular septum, early diastolic pulmonary regurgitation velocity > 2.2 m/s, IVC diameter > 21 mm with reduced inspiratory collapse capacity, right ventricular outflow tract (RVOT) pulmonary ejection acceleration time < 105 ms, reduced right ventricular fractional area change (< 35%); end-systolic area of right atrium > 18 cm^2^, pulmonary artery diameter > aortic root diameter or pulmonary artery diameter > 25 mm [140].

The most accurate and reproducible technique for assessing myocardial function and morphology is cardiac MRI. It allows accurate flow and volume measurements and delayed enhancement of the myocardium.

Computed tomography can provide detailed information that allows direct measurement of pulmonary artery diameter (a diameter of ≥ 30 mm may be considered pathological) and right to left ventricle ratio (RV:LV ratio), which is considered altered if it is ≥ 1. Signs of lung parenchymal involvement, such as ground glass opacity, thickening of interstitial septa or lymphadenopathy, may direct the diagnosis to forms of PH with parenchymal pathogenesis [141].

CT angiography (CTA) provides information about the presence of pulmonary embolism and differentiates PH from acute pulmonary embolism, due to chronic thromboembolic genesis. It also identifies altered perfusion, bronchial artery hypertrophy or vascular abnormalities causing pulmonary shunt.

Modern dual-energy scanners CT (DECT) expand the potential of radiological imaging by providing a spectral analysis of contrast distribution and enabling the reconstruction of virtual iodine distribution maps [142,143]. Iodine perfusion maps have been widely compared with the gold standard for assessing lung perfusion, V/Q scintigraphy, and specific studies of chronic pulmonary embolism in patients with pulmonary hypertension found an excellent correlation coefficient (k = 0.8) [144].

The use of DECT offers additional benefits for patients with impaired GFR. Using virtual monoenergetic imaging (routinely available for every DECT scanner), it is possible to achieve valid contrast-to-noise ratios on pulmonary CTA with dramatically reduced amounts of contrast agent (up to 15 ml/6 g iodine) while maintaining diagnostic accuracy [145,146].

V/Q lung scintigraphy (in conjunction with single-photon emission computed tomography [SPECT] instead of planar scintigraphy) is currently still the technique of choice to exclude chronic pulmonary embolism as the cause of PH, with a negative predictive value of 98%.

Although it is an invasive technique, digital subtraction angiography (DSA) still plays a crucial role in the diagnostic imaging of PH: while other imaging techniques can help to confirm suspicions or identify signs and causes of PH during the clinical examination, DSA is still considered the gold standard for confirming the diagnosis and must be performed according to strict standards and in highly PH specialised centres. Cardiac catheterisation is not limited to the diagnostic phase, as some forms of PH may benefit from percutaneous balloon angioplasty.

## 8. Conclusions

The prevalence of CKD and lung disease is predicted to increase significantly worldwide in the coming years, posing significant economic and societal challenges. The kidney-lung axis provides a critical pathway to regulate body homeostasis and prevent the exacerbation of systemic disease.

These organs share common mechanisms that can lead to the development and progression of both diseases. These include vascular stiffness, neurohormonal activation, tissue hypoxia and abnormal immune cell signalling.

Further research addressing the interrelationships between these organs is needed to better define valid strategies for the treatment of pathological entities directly influenced by the interaction between the kidney and lung, and to individualise treatment decisions.

## Figures and Tables

**Table 1:** Chronic obstructive pulmonary disease classification. Forced expiratory volume, FEV-1.

Stages	FEV 1 Predicted
I (Mild)	≥80%
II (Moderate)	50–79%
III (Severe)	30–49%
IV (Very Severe)	≤30%

**Table 2:** Summary of main characteristics and findings of the studies evaluating the prevalence of renal impairment in COPD patients.

Author	Aim of the Study	Population	Design of the Study	Renal Impairment Prevalence/Renal Outcomes	Conclusion
Incalzi et al., 2010 [12]	Prevalence of CRF in elderly COPD patients	356 COPD, 290 non-COPD They were categorized as having normal renal function (GFR 60 mL/min/1.73 m^2^), concealed CRF (normal serum creatinine and reduced GFR), or overt CRF (increased serum creatinine and reduced GFR)	Observational	20.8% concealed (10.0% without COPD), 22.2% overt (13.4% without COPD)	CRF highly prevalent in patients with COPD
Gjerde et al., 2011 [13]	COPD phenotypes and prevalence of sub-clinical renal failure and the relation with inflammatory markers	433 COPD 233 non-COPD	Cohort	9.6% females with COPD and 5.1% males with COPD patients; GFR < 60	Female sex, higher age, cachexia, and the inflammatory markers sTNF-R1 and NGAL were all independently associated with a higher risk for renal failure in COPD patients
Elmahallawy and Qora 2013 [14]	Frequency of underdiagnosed renal failure	300 COPD and 300 control	Cohort	Normal renal function, concealed, overt in COPD patients, respectively, 54%, 26%, 20%; in controls, 78%, 10%, 12%	CRF is an important comorbidity and estimated GFR is needed for screening
Mapel and Marton 2013 [15]	Prevalence of renal or hepatobiliary disease in COPD patients	2284 COPD and 5959 non-COPD	Cohort	Acute, chronic, and unspecified renal failure 1.40 vs. 0.59, 2.89 vs. 0.79, and 1.09 vs. 0.44, respectively	COPD patients have an increased prevalence of renal, gallbladder, and pancreatic diseases, as well as abnormal renal and hepatic laboratory values. They are also more likely to be prescribed medications with potentially toxic renal or hepatic side effects.
Yoshizawa et al., 2015 [16]	Prevalence of COPD with eGFR based on creatinine and cystatin C levels	108 COPD, 73 non-COPD	Clinical trial	eGFRcr vs. eGFRcys 31% vs. 53% in COPD patients; 8% vs. 15% non-COPD patients	In Japanese COPD patients, renal function should preferably be evaluated based not only on Cr but on Cr in combination with Cys.
Chen and Liao 2016 [17]	Incidence of CKD in COPD patients	7739 COPD patients, 15,478 non-COPD	Case-cohort	HR: 1.61 overall (470.9 vs. 287.52 per 10^4^ person-years)	Patients with COPD have a higher risk of CKD
AbdelHalim and AboElNaga 2016 [18]	Prevalence of CRF in COPD patients	136 COPD, 104 non-COPD	Cohort	19.85% concealed (1.92% non-COPD), 6.66% overt (0% non-COPD)	High prevalence of CRF in COPD patients
Sumida et al., 2017 [19]	Association of reduced lung function with ESRD and CKD	14,946	Prospective cohort	HR for CKD compared to high-normal 1.53, in mixed restrictive 1.42, obstructive 1.15, low-normal 1.08	Reduced lung function is independently associated with CKD progression
Yu et al., 2017 [20]	Association between lung and impaired kidney function	1298 normal renal function, 156 impaired, 4313 normal, 1511 impaired	2 cross-sectional studies	Increased risk for renal impairment below 3.05, both for FEV1 and FVC in both studies	There was a correlation between obstructive lung function and reduced kidney function
Kim et al., 2018 [21]	Impact of lung function in the development of CKD	10,128 subjects	Retrospective cohort	FEV1/FVC < 0.8, incidence of CKD 2.8%	Increased risk of CKD with restricted airflow; a 10% decrease in FEV1/FVC leads to a 35% increase in the development of CKD
Zaigham et al., 2020 [22]	Low lung function early in life and development of CKD in the future	28,025	Prospective cohort	Q1 vs. Q4, HR 1.46 in low FEV1 and HR 1.51 for FVC in men	Low FEV1 and FVC were a risk factor for future incident CKD in men, but not women; FEV1/FVC < 0.7 does not increase the incidence for CKD in both men and women
Suzuki et al., 2020 [23]	Mortality in COPD and CKD	1233 health check-up participants	Cohort	CKDcys 26.1% with AFL vs. 16.2% without AFL	Significantly higher prevalence with CKDcys in AFL, but not with CKDcr
Pelaia et al., 2021 [24]	Incidence of CKD and the rapid decline of eGFR	707 outpatients	Multicenter Observational Cohort	157 (22.2%) patients had CKD at baseline. During a mean follow-up of 52.3 ± 30.2 months, 100 patients developed CKD, and 200 patients showed a rapid reduction of eGFR	COPD patients had a significant worsening of renal function over time
Kim et al., 2021 [25]	Association between obstructive spirometry pattern and incident CKD development	7960 non-CKD patients	Prospective community-based cohort study	Incident CKD developed in 511 subjects (6.4%)	Decreased FEV1/FVC ratio was independently associated with an increased risk of incident CKD development, particularly in people without metabolic syndrome
Boiko et al., 2022 [26]	Renal function parameters as early predictors of kidney damage in patients with hypertension and COPD	88 patients with hypertension and COPD divided into three groups: Group I, 38 patients with hypertension, Group II, 27 patients with hypertension and COPD, Group III, 23 patients with COPD	Cohort	Blood creatinine levels: Group I 88.3 (84.2; 102.7) μmol/l, Group II 99.0 (80.0; 115.0) μmol/l, Group III 84.6 (75.0; 94.2) μmol/l (*p* = 0.008).	Decrease in renal filtration function in all the groups. Negative aggravating effect of COPD on renal function in patients with hypertension

AFL: airflow limitation; CRF: chronic renal failure; CKD: chronic kidney disease; CKDcys: chronic kidney disease based on cystatine; CKDcr: chronic kidney disease based on creatinine; COPD: chronic obstructive pulmonary disorder; eGFR: estimated glomerular filtration rate; eGFRcr: eGFR based on creatinine; eGFRcys: eGFR based on cystatine; ESRD: end-stage renal disease; FEV1: forced expiratory volume in the first second; FVC: forced vital capacity; HR: hazard ratio; NGAL: neutrophil gelatinase-associated lipocalin; OR: odds ratio; sTNF-R1: soluble tumor necrosis factor receptor 1.

**Table 3:** Classification of apnoea-hypopnea index (AHI) for sleep apnoea classification [54].

Sleep Apnoea Classification	Episodes per Hour of Sleep
Mild	5 to 15
Moderate	15 to 30
Severe	>30

## Data Availability

Not applicable.
